# Reply to Xiao and Imada: Prosociality across group boundaries is possible without partner choice

**DOI:** 10.1073/pnas.2613719123

**Published:** 2026-06-08

**Authors:** Kasper Otten

**Affiliations:** ^a^Asylum and Migration Group, WODC Research and Data Centre, The Hague 2500 EH, The Netherlands; ^b^ Erasmus School of Social and Behavioural Sciences, Erasmus University Rotterdam, Rotterdam 3000 DR, The Netherlands

When benefitting one’s ingroup comes at the cost of an outgroup, these groups are said to be negatively interdependent. In such situations, ingroup prosociality can go together with outgroup hostility (1). In the absence of negative interdependence, I showed that ingroup prosociality tends to go together with outgroup prosociality instead of hostility (2). Across six cross-societal datasets where people make independent decisions regarding ingroups and outgroups, those who are prosocial toward ingroups also tend to be prosocial toward outgroups, albeit at somewhat lower levels. Xiao and Imada (3) acknowledge this co-occurrence of ingroup and outgroup prosociality, but suggest that it may not manifest in practice because people avoid interactions with outgroups. They therefore argue that this co-occurrence requires cross-group partner choice. I appreciate this insightful comment and recognize the importance of cross-group partner choice. However, I do not think it is required for the co-occurrence of ingroup and outgroup prosociality, for two related reasons.

First, in today’s multicultural and interconnected societies, it is impossible to avoid cross-group interactions. For example, at the workplace, people often must interact with colleagues from different ethnic, religious, or political backgrounds. At schools, people are placed together with classmates from different social, cultural, and economic backgrounds. And in many daily situations, people encounter strangers from different backgrounds in public transport, neighborhoods, and online environments. People will thus have to interact with both ingroups and outgroups even if they themselves would prefer interacting only within their ingroup. In the datasets that I analyzed, people also did not choose to interact with outgroups; they were paired with ingroups and outgroups by the experimenter or survey design. Yet, the co-occurrence of ingroup and outgroup prosociality consistently appeared (2).

Second, having to interact with outgroups may lead people to be more willing to interact with outgroups in the future. There is a large body of evidence on intergroup contact theory suggesting that exposure to outgroups can reduce outgroup prejudice and increase the willingness to interact with outgroups, particularly when the exposure was positive (4, 5). Hence, even when people are initially not eager to interact with outgroups, positive experiences in situations where they are required to interact with outgroups may lead them to be more open to cross-group interactions. The datasets I analyzed also show that forced interactions with outgroups can indeed be positive. Such positive experiences may thus have spill-over effects for the willingness to interact with outgroups in the future.

Altogether, I think that cross-group partner choice makes the co-occurrence of ingroup and outgroup prosociality more likely, but it is not required. Even without choosing interaction partners, people are often prosocial toward outgroups when interactions occur in the absence of negative interdependence. Thus, in the many real-world situations where cross-group encounters are unavoidable, prosociality across group boundaries is still possible. These positive outgroup interactions may even increase future willingness for cross-group partner choice. Establishing prosociality across group boundaries remains challenging, but the present findings suggest it is feasible when negative interdependence is absent.
